# Chromosomal Mechanisms of Colistin Resistance in Clinical Isolates of Carbapenem-Resistant *Klebsiella pneumoniae* from a Tunisian Tertiary-Care Hospital

**DOI:** 10.3390/idr18030042

**Published:** 2026-05-01

**Authors:** Zaineb Hamzaoui, Hajer Kilani, Alain Ocampo-Sosa, Sana Ferjani, Elaa Maamar, Lamia Kanzari, Ahmed Fakhfakh, Amel Rehaiem, Luis Martínez-Martínez, Ilhem Boutiba Ben Boubaker

**Affiliations:** 1 LR99ES09, Faculty of Medicine of Tunis, University of Tunis El Manar, Tunis 1007, Tunisia; hajerkilani@yahoo.fr (H.K.); sana.ferjani@rns.tn (S.F.); elaa.maamar@fst.utm.tn (E.M.); lamia.kanzari@fmt.utm.tn (L.K.); ahmed81fakhfakh@gmail.com (A.F.); amel.rehaiem@fmt.utm.tn (A.R.); ilhem.boutiba@rns.tn (I.B.B.B.); 2 Microbiology Service, University Hospital Marqués de Valdecilla, 39008 Santander, Spain; alain.ocampo@scsalud.es; 3 Marqués de Valdecilla Research Institute (IDIVAL), 39008 Santander, Spain; 4 Center for Biomedical Research in Infectious Diseases Network (CIBERINFEC), Instituto de Salud Carlos III, 28020 Madrid, Spain; luis.martinez.martinez.sspa@juntadeandalucia.es; 5 Laboratory of Microbiology, Charles Nicolle Hospital, Tunis 1006, Tunisia; 6 Maimonides Biomedical Research Institute of Cordoba (IMIBIC), 14004 Cordoba, Spain; 7 Clinical Unit of Microbiology, University Hospital Reina Sofía, 14004 Cordoba, Spain; 8 Department of Agricultural Chemistry, Soil Science and Microbiology (Microbiology Area), University of Cordoba, 14014 Cordoba, Spain

**Keywords:** colistin resistance, *mgrB*, *phoP*, *phoQ*, carbapenemase, porins, multidrug resistance, virulence factors

## Abstract

Background/Objectives: Carbapenem-resistant *Klebsiella pneumoniae* (CRKP) is a major nosocomial pathogen. Although newer agents have reduced colistin use in high-income countries, this polymyxin remains important in many low- and middle-income settings. Colistin resistance in *K. pneumoniae* is most commonly associated with chromosomal alterations affecting the MgrB–PhoPQ pathway, or with plasmid-mediated *mcr* genes. This study aimed to investigate chromosomally mediated colistin resistance in CRKP clinical isolates from a Tunisian tertiary hospital. Methods: Between 2010 and 2015, 317 non-duplicate CRKP isolates were collected at Charles Nicolle Hospital, Tunis. Colistin MICs were determined by broth microdilution. Phenotypic tests and PCR characterized carbapenemases, extended-spectrum β-lactamases, AmpC, plasmid-mediated quinolone resistance, *mcr* and virulence genes. Porins (OmpK35/OmpK36) and the *mgrB*, *phoP* and *phoQ* loci were analyzed by SDS-PAGE and sequencing. Clonal relatedness was assessed by ERIC-PCR and multilocus sequence typing. We additionally compared colistin-resistant isolates with a panel of colistin-susceptible CRKP controls and assessed phenotypic stability after serial passages without colistin. Results: Five isolates (1.6%) were colistin-resistant. All were multidrug-resistant, produced OXA-48, and two also carried NDM-1. The isolates belonged to five distinct sequence types, including high-risk clones (ST11, ST101, ST147). No *mcr* genes were detected. Four isolates carried disruptive mutations in *mgrB*, and the remaining strain harbored inactivating mutations in both *phoP* and *phoQ* with an intact *mgrB*. Truncating alterations in PhoP/PhoQ and frequent loss or truncation of OmpK35/OmpK36 were observed. No *mgrB*/*phoP*/*phoQ* alterations were detected among colistin-susceptible controls, and colistin MICs remained stable after 7 days of drug-free passaging. Conclusions: In Tunisian CRKP, colistin resistance was associated with chromosomal alterations, predominantly involving disruption of the MgrB–PhoPQ pathway, in the absence of *mcr* genes. These mechanisms in both high-risk and emerging sequence types underscore the adaptability of CRKP and the need for surveillance where colistin remains an important therapeutic option.

## 1. Introduction

Antimicrobial resistance (AMR) is a global public health crisis, and carbapenem-resistant *Klebsiella pneumoniae* (CRKP) epitomizes the problem [1]. Over the past two decades, CRKP has spread throughout hospitals, aided by acquisition of carbapenemase genes and loss or alteration of major porins that impede β-lactam entry. Patients with CRKP infections often have few therapeutic options, so polymyxins such as colistin (polymyxin E) are used as last-resort drugs [2].

Colistin’s bactericidal action involves binding to the negatively charged lipid A component of lipopolysaccharide (LPS), displacing Mg^2+^/Ca^2+^ ions and disrupting the outer membrane [3].

For many years, colistin has been used as a last-resort drug against multidrug-resistant Gram-negative pathogens, including carbapenem-resistant *K. pneumoniae* (CRKP). More recently, however, several new treatment options have become available for CRKP, such as β-lactam/β-lactamase inhibitor combinations and cefiderocol, which have displaced colistin as a first-line option in many high-income settings [4]. Nevertheless, in many low- and middle-income countries, including North African hospitals, access to these newer agents remains limited and colistin is still widely used in routine practice.

Chromosomal colistin resistance results from mutations that dysregulate lipid A modification pathways. In *K. pneumoniae*, the PhoPQ and PmrAB two-component systems (TCSs) sense environmental signals and control expression of the pmrCAB and arnBCADTEF (pmrHFIJKLM) operons responsible for adding 4-amino-4-deoxy-L-arabinose (L-Ara4N) or PEtN to lipid A. Disruptive mutations in the *mgrB* gene (a 47-amino acid negative regulator of PhoPQ) remove feedback inhibition, leading to constitutive activation of PhoPQ and PmrAB and up-regulation of LPS-modifying enzymes. Similarly, activating mutations in *phoP*/*phoQ*, *pmrA*/*pmrB*, or *crrA*/*crrB* can up-regulate these operons; the net effect is lipid A modification and reduced colistin binding. These chromosomal changes are stable and cannot be transferred horizontally, but they can accumulate within clonal lineages [5].

Beyond colistin, CRKP often carries multiple β-lactamases, aminoglycoside-modifying enzymes and plasmid-borne quinolone resistance genes, contributing to multidrug-resistance profiles. The combination of carbapenemase production, porin loss, and colistin-resistance mechanisms produces extensively drug-resistant or pandrug-resistant strains that are difficult to treat.

Tunisia has reported both plasmid-mediated *mcr* genes [6,7] and chromosomal *mgrB* mutations [8] among clinical *K. pneumoniae* isolates, highlighting the local public health importance of this resistance.

This study aimed to investigate the molecular and phenotypic basis of colistin resistance in CRKP clinical isolates recovered from a Tunisian university hospital. We focused on chromosomal mechanisms involving the MgrB–PhoPQ regulatory pathway, while acknowledging that other two-component systems (such as PmrAB and CrrAB) and genome-wide changes may also contribute to colistin resistance, but these were not explored here due to resource constraints. In addition, because colistin resistance in CRKP usually emerges on a background of broad β-lactam resistance, we also characterized outer membrane porin alterations to better define the multidrug-resistant profile of these isolates.

## 2. Materials and Methods

### 2.1. Study Design

Between 2010 and 2015, all clinical CRKP strains were collected from various specimens at the Microbiology Laboratory of Charles Nicolle Hospital in Tunis, Tunisia. Among the collected CRKP isolates, colistin resistance was assessed by determining the minimum inhibitory concentration (MIC) using the broth microdilution method, following the European Committee on Antimicrobial Susceptibility Testing (EUCAST) guidelines [9]. All subsequent phenotypic assays, molecular characterizations, and genetic analyses were performed on the colistin-resistant CRKP strains.

### 2.2. Species Identification and Antimicrobial Susceptibility Testing

Bacterial species were identified using the API20E system (BioMérieux, Marcy-l’Etoile, France) and confirmed by Matrix-Assisted Laser Desorption Ionization-Time of Flight (MALDI-TOF) (Bruker Daltonics GmbH, Bremen, Germany).

Initially, susceptibility to amoxicillin, amoxicillin–clavulanic acid, cefoxitin, ceftazidime, cefotaxime, cephalotin, cefepime, ertapenem, imipenem, aztreonam, amikacin, tobramycin, netilmicin, nalidixic acid, ofloxacin, ciprofloxacin, fosfomycin, tetracycline, minocycline, and tigecycline was determined by disk diffusion using Mueller-Hinton agar (Bio-Rad, Marnes-la-Coquette, France), according to EUCAST guidelines [9].

MICs of ertapenem, imipenem, and meropenem were determined using E-test strips (BioMérieux, Marcy-l’Étoile, France), while MICs of amoxicillin–clavulanic acid, cefepime, cefotaxime, ceftazidime, and tigecycline were assessed using the Vitek 2 system (BioMérieux, Marcy-l’Étoile, France). All results were interpreted according to EUCAST breakpoints and guidelines [9].

### 2.3. Phenotypic Assays

The modified Hodge test (MHT) was performed according to CLSI guidelines [10] using an ertapenem disk (10 µg). In addition, the Carbapenem Inactivation Method (CIM) was used to detect carbapenemase activity in all isolates, as previously described [11].

Screening for class A carbapenemases was performed using the phenylboronic acid (PBA) method. Inhibition zones obtained with imipenem disks with or without PBA (0.05 M solution) were compared after overnight incubation; an increase of ≥5 mm in the presence of PBA was interpreted as positive for class A carbapenemase production [12].

Screening for class B carbapenemases was performed by comparing inhibition zones around imipenem disks with or without ethylenediaminetetraacetic acid (EDTA, 10 µL of a 0.5 M solution) [13]. An increase in the inhibition zone of >7 mm in the presence of EDTA was considered indicative of metallo-β-lactamase production.

Phenotypic detection of extended-spectrum β-lactamases (ESBLs) was carried out by the double-disk synergy test (DDST) according to CLSI recommendations [10]. AmpC producers were defined as isolates showing a negative ESBL phenotype while being resistant to both cefoxitin and amoxicillin–clavulanic acid.

### 2.4. Chromosomal Modifications in LPS Biosynthesis

A targeted molecular analysis was conducted to investigate chromosomal alterations in the LPS biosynthesis pathway, focusing on the *mgrB*, *phoP*, and *phoQ* genes. These genes were amplified by polymerase chain reaction (PCR) using gene-specific primers under optimized thermal cycling conditions. The resulting PCR products were purified and subjected to Sanger sequencing.

As a comparator set, we screened 19 carbapenem-resistant but colistin-susceptible *K. pneumoniae* isolates collected during the same study period at the same hospital (previously described elsewhere [14]). The full coding regions of *mgrB*, *phoP* and *phoQ* were amplified and Sanger-sequenced using the same workflow as for the colistin-resistant isolates.

DNA and deduced amino acid sequences were analyzed using the VECTOR NTI (Invitrogen, Carlsbad, CA, USA) and compared with the reference genome of the colistin-susceptible *K. pneumoniae* ATCC 13883 strain (GenBank accession number NZ_JOOW00000000).

### 2.5. Stability of the Colistin-Resistant Phenotype

To assess the stability of the colistin-resistant phenotype in the absence of selective pressure, each colistin-resistant isolate was serially passaged daily on colistin-free medium for seven consecutive days. Briefly, a single colony from each isolate was subcultured every 24 h onto fresh antibiotic-free plates and incubated at 37 °C. After the seventh passage, colistin MICs were re-determined by broth microdilution under the same conditions as at baseline, and compared with the initial MICs. Phenotypic stability was defined as an unchanged MIC (or variation within one two-fold dilution), consistent with previously used approaches for assessing colistin-resistance stability. In addition, to assess genetic stability of the primary chromosomal determinant, the *mgrB* locus was re-amplified and Sanger-sequenced after day 7 for each isolate, using the same primers and sequencing workflow as at baseline. Day-7 *mgrB* sequences were aligned against the corresponding baseline sequences to detect any reversion or secondary changes.

### 2.6. Molecular Detection of Resistance Genes

Multiplex PCR assays were conducted to identify the most commonly encountered carbapenemase genes, including class A (*bla_KPC_* and *bla_GES_*), class B (*bla_IMP_* and *bla_VIM_*), and class D (*bla_OXA-48-like_*), following previously established protocols [15]. The detection of the *bla_NDM_* gene was carried out as described in other studies [16].

In addition, screening was performed for ESBL genes (*bla_CTX-M_*, *bla_TEM_*, *bla_SHV_*, *bla_VEB_*, *bla_GES_*, and *bla_PER_*) and plasmid-mediated AmpC β-lactamase genes (*bla_CIT_*, *bla_MOX_*, *bla_FOX_*, *bla_EBC_*, *bla_DHA_*, and *bla_ACC_*) [15]. Furthermore, plasmid-mediated quinolone resistance (PMQR) genes (*qnrA*, *qnrB*, *qnrC*, *qnrD*, *qnrS*, *qepA*, *oqxAB*, and *aac(6′)-Ib-cr*) were investigated [17], together with aminoglycoside resistance genes (*acc(6′)-Ib*, *aac(3′)-Ia*, *aac(3′)-IIa*, *aac(3′)-IVa*, *aph(3′)-Ia*, *aph(3′)-IIa*, *aph(3′)-VIa*, *ant(2″)-Ia*) [18].

The presence of plasmid-mediated colistin resistance was assessed through PCR amplification of *mcr* genes using specific primers for each target [19].

The PCR products were subsequently purified and sequenced, and the resulting DNA sequences were compared with reference nucleotide sequences available in the GenBank database.

### 2.7. Detection of Virulence-Associated Genes in K. pneumoniae

The virulence profile was analyzed by PCR to check for the presence of nine genes linked to virulence in *K. pneumoniae*, namely capsular serotype K1 and hypermucoviscosity phenotype (*magA*), allantoin metabolism (*allS*), regulator of mucoid phenotype A (*rmpA*), iron system capture (*iroN*), capsular serotype K2 and hypermucoviscosity phenotype (*cps*), adhesion type 3 fimbriae (*mrkD*), iron transport and phosphotransferase function (*kfu*), siderophore (*entB*), and siderophore yersiniabactin (*ybtS*) [20].

### 2.8. Molecular Epidemiology and Phylogenetic Analysis

Enterobacterial Repetitive Intergenic Consensus (ERIC) PCR was used as described previously [21] to assess the genetic relatedness of the *K. pneumoniae* strains. This method targets repetitive DNA sequences within the bacterial genome, providing insight into clonal diversity.

Multilocus sequence typing (MLST) was performed using a previously standardized MLST protocol. The scheme used the following seven housekeeping genes: *gapA*, *infB*, *mdh*, *pgi*, *phoE*, *rpoB*, and *tonB* [22]. The allelic profile was summarized by assigning a sequence type (ST) via a web database (https://bigsdb.pasteur.fr/klebsiella/, accessed on 30 March 2016).

To contextualize the five colistin-resistant isolates, a comparator panel of 19 colistin-susceptible carbapenem-resistant *K. pneumoniae* isolates (CS-CRKP01 to CS-CRKP19) was included in the phylogenetic analysis [14]. Phylogenetic relationships were inferred from concatenated nucleotide sequences of the seven MLST housekeeping genes (*gapA*, *infB*, *mdh*, *pgi*, *phoE*, *rpoB*, and *tonB*). Sequences were aligned using ClustalW in MEGA (version 11.0.13), and a maximum-likelihood tree was generated under the Tamura–Nei model; branch lengths represent the number of substitutions per site. All positions containing gaps and missing data were eliminated from the final dataset. The resulting tree was visualized and annotated using iTOL (v6, https://itol.embl.de/, accessed on 30 November 2025). A presence/absence heatmap of selected β-lactamase genes, plasmid-mediated quinolone resistance (PMQR) determinants, and virulence-associated genes (as determined by our screening pipeline) was displayed alongside the tree.

### 2.9. Characterization of Outer Membrane Proteins and Sequencing of ompK35 and ompK36 Genes

Outer membrane protein preparations were obtained by sonication of bacterial cells cultured in Mueller-Hinton broth, followed by selective solubilization of the cytoplasmic components with 2% sodium lauroyl-sarcosynate and ultracentrifugation. The samples were then boiled, loaded onto 11.0% sodium dodecyl sulfate-polyacrylamide gels, and stained with Coomassie blue [23]. Reference controls included *K. pneumoniae* SD8 (positive control expressing OmpK35 and OmpK36 [24]) and *K. pneumoniae* CSUB10R (negative control lacking detectable OmpK35 and OmpK36 [25]). In addition, *K. pneumoniae* KCTC2242 and *K. pneumoniae* NTUH-K2044 were also loaded on the SDS–PAGE gels as porin-specific reference controls (PC-K35 and PC-K36, respectively) to validate band assignment. The *ompK35* and *ompK36* genes were amplified, sequenced, and compared with the *ompK* gene sequences of *K. pneumoniae* KCTC2242, which produces OmpK35 and lacks OmpK36 (NCBI accession number CP002910), and *K. pneumoniae* NTUH-K2044, which produces OmpK36 and lacks OmpK35 (NCBI accession number AP006725), using VECTOR NTI (v11.5, Invitrogen, Carlsbad, CA, USA) [26].

Putative housekeeping (σ70/RpoD) promoter signals upstream of *ompK35* were predicted in silico using BPROM (SoftBerry) (SoftBerry Inc., Mount Kisco, NY, USA; available online: http://www.softberry.com/berry.phtml?topic=bprom (accessed on 23 December 2025)). For each analyzed sequence, an upstream fragment of *ompK35* was submitted to BPROM using default parameters (threshold 0.20), and the predicted −35/−10 elements and transcription start site (TSS) positions were recorded.

Densitometric analysis. Band intensities were quantified by densitometry using Fiji (ImageJ; v1.54s11; National Institutes of Health, Bethesda, MD, USA) [27]. For each lane, the OmpK35 and OmpK36 bands were background-subtracted and normalized to the OmpA band (loading control). Normalized values were expressed relative to the porin-specific positive controls (PC-K35 for OmpK35 and PC-K36 for OmpK36; control lanes set to 100%). Marker lanes were excluded from the analysis.

## 3. Results

### 3.1. Prevalence of Colistin Resistance in CRKP Isolates and Antimicrobial Resistance Profile

A total of 317 nonredundant clinical CRKP strains were collected from various wards of Charles Nicolle Hospital between 2010 and 2015. Of these, 5 isolates exhibited resistance to colistin, accounting for 1.6% of the total isolates. The colistin-resistant isolates were primarily recovered from the Intensive Care Unit (ICU) (*n* = 4), with one isolate from the Orthopedics ward. They were recovered from different specimen types: pulmonary (*n* = 2), catheter (*n* = 2), and wound (*n* = 1).

The patients’ ages ranged from 24 to 84 years, with 4 of the 5 patients being male. They were admitted either for respiratory distress or polytrauma. Three of these patients were treated during hospitalization with a combination of colistin and imipenem. In addition, combinations of different antibiotic families, namely aminoglycosides (such as amikacin or gentamicin) and fluoroquinolones (mainly ciprofloxacin), in association with imipenem, were used. Despite antibiotic treatment, 1 patient died during hospitalization (Table 1).

All isolates were confirmed as *K. pneumoniae* by both API20E and MALDI-TOF. Antimicrobial susceptibility testing by disk diffusion revealed resistance to all tested penicillins and cephalosporins, as well as fluoroquinolones and gentamicin; two isolates were non-susceptible to tigecycline based on MICs obtained with the VITEK2 system (Table 2).

E-test results confirmed resistance to ertapenem for all five isolates, with MICs ranging from 2 to >32 mg/L. Two isolates were resistant to imipenem (MICs 2–24 mg/L), and three were resistant to meropenem (MICs 1–24 mg/L). All isolates exhibited colistin resistance, with MICs ranging from 8 to 32 mg/L. Furthermore, all isolates tested positive for carbapenemase production using both MHT and CIM. EDTA-based synergy testing was positive for the two *bla*_NDM-1_-producing isolates and negative for the remaining strains, whereas no isolate showed a ≥5-mm increase in imipenem inhibition zone in the presence of phenylboronic acid, in keeping with the absence of KPC-type carbapenemases (Table 2).

### 3.2. Characterization of Antimicrobial Resistance Genes

**Figure 1:**
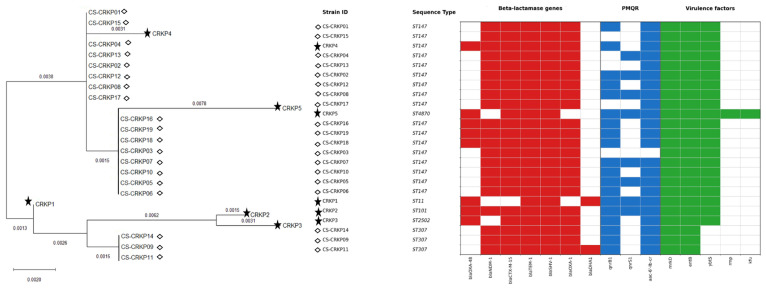
Phylogenetic tree of colistin-resistant *K. pneumoniae* isolates constructed in MEGA (v11.0.13) after multiple sequence alignment with ClustalW. The tree was inferred using the maximum-likelihood approach under the Tamura–Nei substitution model; branch lengths represent the number of nucleotide substitutions per site (scale bar shown). The dendrogram displays the genetic relatedness of the five study isolates (★ CRKP1–CRKP5) and the comparator strains (◇ CS-CRKP01–CS-CRKP19). Sequence types (STs) are indicated alongside each strain. The heatmap aligned with the tree summarizes the presence/absence of acquired β-lactamase genes (red), plasmid-mediated quinolone resistance (PMQR) genes (blue), and virulence-associated genes (green); white cells indicate absence of the corresponding gene.

Molecular fingerprints grouped the 5 isolates into 4 distinct patterns: A (2 strains), B (1 strain), C (1 strain), and D (1 strain). Furthermore, the MLST results revealed the presence of 5 different sequence types (STs): ST11, ST101, ST2502, ST147, and ST4870 (Figure 1).

The phylogenetic analysis, including the colistin-susceptible comparator panel, showed that the five colistin-resistant CRKP isolates did not form a single monophyletic cluster. CRKP2 and CRKP3 clustered together, whereas CRKP1 branched separately from the remaining isolates. CRKP4 and CRKP5 were placed within the ST147 background alongside multiple colistin-susceptible isolates, with CRKP5 showing a longer branch length, suggesting greater divergence (Figure 1).

All strains carried the *bla_OXA-48_* gene, and *bla_NDM-1_* was additionally detected in two strains (CRKP4 and CRKP5). The extended-spectrum β-lactamase (ESBL) *bla_CTX-M-15_* was present in all strains except for CRKP1, which harbored the AmpC β-lactamase *bla_DHA-1_*. Other β-lactamase genes identified included *bla_TEM-1_*, *bla*_SHV-1_, and *bla*_OXA-1_, along with plasmid-mediated quinolone resistance genes *qnrS1*, *qnrB1*, and *aac(6′)-Ib-cr* in certain strains. No aminoglycoside resistance genes were detected. No plasmid-mediated colistin resistance genes (*mcr*) were detected. Three virulence-associated genes, *mrkD*, *entB*, and *ybtS*, were present in all strains, while *rmp* and *kfu* were exclusively found in CRKP3 (Figure 1).

### 3.3. Porin Expression and ompk35/ompk36 Alterations

SDS-PAGE analysis showed that all strains expressed a ~32 kDa protein corresponding to the structural protein OmpA but failed to express a full complement of porins compared to the positive controls *K. pneumoniae* KCTC2242 (expressing OmpK35 but lacking OmpK36) and *K. pneumoniae* NTUH-K2044 (expressing OmpK36 but lacking OmpK35) (NCBI accession numbers CP002910 and AP006725, respectively) (Supplementary Figure S1).

Notably, three isolates, CRKP1, CRKP2, and CRKP3 (belonging to pulsotypes B/ST11, C/ST101 and D/ST2502, respectively), lacked the ~39 kDa band corresponding to the major porin OmpK35.

Sequence analysis of *ompK35* in CRKP1 revealed the insertion of an ISKpn14 element (~0.78 kb) upstream of the start codon, spanning positions −852 to −72 relative to the A of the ATG start codon, and oriented in the same direction as *ompK35*. This IS1-family insertion, located within the putative promoter region, is likely responsible for the absence of detectable OmpK35 in the outer membrane protein profile (Table 3; Supplementary Figure S2).

To assess the potential transcriptional impact of the ISKpn14 insertion, promoter prediction was performed using BPROM. In the reference strain (*K. pneumoniae* NTUH-K2044) upstream region (460 bp), BPROM predicted one σ70-like promoter with a strong score (LDF 7.19), with −35/−10 elements at positions 146 (TTGCCG; score 55) and 168 (ATAAATAAT; score 34), respectively, and a predicted TSS at position 182. In contrast, analysis of the corresponding CRKP1 upstream sequence (460 bp) yielded a much weaker promoter signal (LDF 0.97), with a very low −35 score (position 148; score 4) despite a detectable −10 motif (position 172; TTGTACAGT; score 47).

In CRKP2 (pulsotype C, ST101) and CRKP3 (pulsotype D, ST2502), sequencing of *ompK35* followed by alignment with the reference strain *K. pneumoniae* NTUH-K2044 identified multiple alterations. These included point mutations (C156T in CRKP3), deletions [a six-nucleotide deletion (5′-CACCAA-3′) at position 137 in CRKP2 and a single-nucleotide deletion at position 184 in CRKP3], and a single-nucleotide insertion at position 166 in CRKP2. Collectively, these changes introduced premature stop codons, leading to truncated OmpK35 proteins of 314 amino acids in CRKP2 and only 62 amino acids in CRKP3, compared with the full-length 359-amino acid protein in the reference strain (Table 3; Supplementary Figure S1).

In the CRKP4 strain (pulsotype A, ST147), sequence analysis of the *ompK36* gene revealed several point mutations as well as the insertion of a nine-nucleotide sequence [5′-CTGTCTCCT-3′] located at position 550 downstream of the start codon. These genetic alterations result in a protein sequence that diverges from that of the reference strain *K. pneumoniae* KCTC2242. Consistent with these findings, no detectable OmpK36 band was observed in SDS-PAGE profiles (Table 3; Supplementary Figure S1).

In the CRKP5 strain (pulsotype B, ST4870), two major mutations were identified: the insertion of a guanine at position 65 and a thymine at position 129, both downstream of the start codon. These frameshift events introduce a premature stop codon, leading to the production of a truncated protein of only 22 amino acids instead of the full-length 365-residue OmpK36 (Table 3; Supplementary Figure S1).

To provide objective support for the qualitative SDS-PAGE interpretation, we performed densitometric quantification of OmpK35 and OmpK36 bands. After normalization to OmpA, CRKP1, CRKP2, and CRKP3 showed detectable OmpK36 signal with markedly reduced/undetectable OmpK35, whereas CRKP4 and CRKP5 showed detectable OmpK35 signal with markedly reduced/undetectable OmpK36. The normalized values and percent expression relative to the porin-specific positive controls are reported in Supplementary Table S1.

### 3.4. Chromosome-Mediated Colistin Resistance: Mutations in mgrB, phoP and phoQ

**Figure 2:**
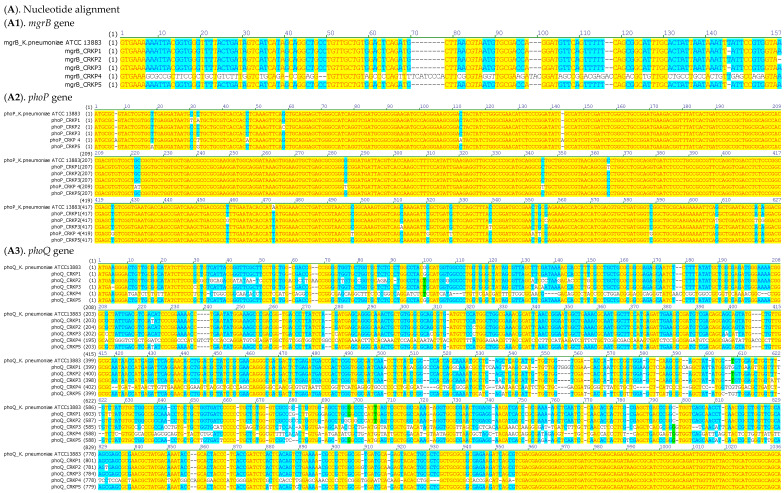

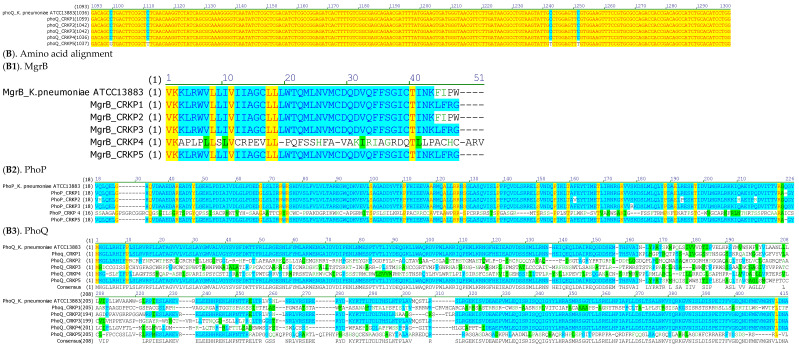
Multiple sequence alignments of *mgrB*, *phoP*, and *phoQ* genes and their encoded proteins in colistin-resistant CRKP strains. (**A**). Nucleotide sequence alignments of (**A1**) *mgrB*, (**A2**) *phoP*, and (**A3**) *phoQ* genes in colistin-resistant CRKP isolates compared to the *K. pneumoniae* ATCC 13883 reference strain. (**B**). Corresponding amino acid sequence alignments of (**B1**) MgrB, (**B2**) PhoP, and (**B3**) PhoQ proteins. Conserved regions are indicated by colored boxes, while mismatches, insertions, and deletions highlight genetic variations. Yellow indicates conserved positions across all isolates, blue highlights variable sites (at least one sequence with a difference), and dashes represent insertions/deletions. Colored letters correspond to the default residue-coloring scheme applied by the alignment software and do not encode additional experimental information.

Sequencing of colistin resistance-associated genes (*mgrB*, *phoP*, and *phoQ*) revealed multiple alterations across the five CRKP isolates (Table 4; Figure 2).

Analysis of the *mgrB* gene revealed distinct alterations among the CRKP isolates. Three isolates (CRKP1, CRKP3, and CRKP5) carried a deletion at nucleotide 132, resulting in frameshift events and altered protein sequences. CRKP4 exhibited multiple point mutations and nucleotide deletions (ΔA42, ΔCC48–49), leading to a premature stop codon at amino acid 19. In contrast, CRKP2 showed no detectable alterations, maintaining an intact *mgrB* sequence. Sequence-based comparison of *mgrB* further supported these observations. CRKP4 carried the most disruptive *mgrB* changes, consistent with a markedly altered sequence relative to the reference, whereas CRKP2 maintained an intact *mgrB* allele. The remaining isolates showed distinct *mgrB* profiles driven mainly by the single-nucleotide deletion at position 132.

Comparative analysis of the *phoP* gene revealed heterogeneous mutational profiles among the five CRKP isolates. CRKP1 carried three nucleotide changes (C29T, C31A, C363T) leading to amino acid substitutions. CRKP2 showed mutations (G57C, T457G, G554C), also resulting in amino acid substitutions without evidence of truncation. CRKP3 harbored three substitutions (A471T, C510A, and C537T) predicted to alter the amino acid sequence. In contrast, CRKP4 exhibited a complex mutation pattern (insertion of nucleotide A at position 7, C32T, C47A, C112A, insertion of nucleotide A at position 143), generating a premature stop codon at amino acid 7, consistent with a truncated non-functional protein. Finally, CRKP5 displayed two substitutions (T17A and C31G) affecting the N-terminal region.

Overall, the MLST-based phylogeny (Figure 1) indicates that these alterations occur across distinct genetic backgrounds rather than within a single clonal expansion.

Analysis of the *phoQ* gene revealed multiple disruptive events across the studied CRKP isolates. CRKP1 carried combined mutations (C219T, InsA486, InsGC498-499), which introduced a premature stop codon at amino acid 175. CRKP2 harbored an extensive set of substitutions and indels (T32G, C35G, A36C, T37A, T38G, ΔC48, InsA58, InsAA65-66, ΔC91) that generated a premature stop codon at amino acid 41, predicting a severely truncated PhoQ protein. CRKP3 exhibited frameshift-inducing mutations (ΔA5, G29C, T130A, ΔC611), leading to an altered protein sequence. The most severe alterations were found in CRKP4, with a cluster of substitutions and deletions (C10T, T11G, G12A, T14C, G15T, G17T, C19T, A20T, ΔT34, ΔGCC105-106-107) that produced a premature stop codon at amino acid 4, consistent with complete loss of function. Finally, CRKP5 carried multiple substitutions and indels (C219T, C512A, C517A, ΔG527, InsT538), resulting in a premature stop codon at amino acid 207.

We further examined whether colistin MIC levels tracked with the predicted severity of *mgrB*/*phoP*/*phoQ* alterations. While the highest MIC (32 mg/L) occurred in an isolate carrying a truncating event in PhoQ in addition to other pathway changes, the small number of isolates and the presence of disruptive alterations across all strains preclude any robust correlation analysis. An integrated summary of MIC values, mutation type, and predicted functional impact is provided in Table 5.

To determine whether the detected *mgrB*/*phoP*/*phoQ* alterations could reflect background polymorphisms rather than resistance-associated events, we analyzed 19 colistin-susceptible CRKP isolates from the same hospital and time period. None of these comparator isolates showed disruptive changes (insertions/deletions, frameshifts, premature stop codons, or IS-mediated interruptions) in *mgrB*, *phoP* or *phoQ*, and no non-synonymous substitutions were detected in the sequenced regions.

Overall, gene-level comparisons of *mgrB/phoP/phoQ* sequences highlighted marked heterogeneity among the five colistin-resistant isolates, with CRKP4 consistently showing the most disruptive predicted changes. In contrast, none of the 19 colistin-susceptible comparator isolates displayed disruptive events (insertions/deletions, frameshifts, premature stop codons, or IS-mediated interruptions) in *mgrB*, *phoP* or *phoQ*.

### 3.5. Stability of Colistin Resistance After Serial Passaging Without Antibiotic Pressure

Following seven consecutive daily passages on colistin-free medium, colistin MIC values remained unchanged for all five colistin-resistant CRKP isolates compared with baseline. No isolate showed a decrease in MIC toward colistin susceptibility. Consistently, repeat Sanger sequencing of *mgrB* after day 7 showed no nucleotide changes compared with baseline for any isolate.

## 4. Discussion

In this study, five colistin-resistant CRKP isolates were identified among 317 non-duplicate clinical strains collected over a five-year period in a Tunisian hospital.

These colistin-resistant isolates accounted for 1.6% of all CRKP strains and were mostly recovered from intensive care unit (ICU) patients. Although this prevalence is relatively low, it remains clinically concerning. Reported colistin resistance rates vary globally, with higher values in some regions, such as 39.1% in Nigeria, 22.5% in Kenya, and 19.2% in parts of Asia, while many European and North American countries report rates below 5% [28,29,30]. Our findings align more closely with these lower global estimates. However, since our isolates were collected between 2010 and 2015, direct comparison with more recent prevalence data should be interpreted cautiously.

Colistin resistance is primarily driven by the burden of healthcare-associated infections and extensive use of polymyxins, particularly in ICUs [31]. Resistance may also emerge spontaneously through chromosomal mutations or be acquired via horizontal gene transfer, even in the absence of prior colistin exposure [32]. In our study, patients infected with colistin-resistant CRKP ranged in age from 24 to 84 years, were predominantly male, and were admitted for severe conditions such as respiratory distress and polytrauma, clinical scenarios often requiring broad-spectrum antibiotics and invasive procedures, both risk factors for acquiring MDR infections.

Combination therapies incorporating colistin and imipenem, along with aminoglycosides and fluoroquinolones, were commonly used in our cohort, reflecting current clinical practices aimed at enhancing treatment efficacy against CRKP [33]. Although in vitro and observational studies support the synergistic potential of colistin-imipenem combinations [34], therapeutic success remains variable. In our series, only one patient died during hospitalization, suggesting a possible benefit of combination therapy, though clinical outcomes remain unpredictable in critically ill patients.

Colistin-resistant isolates in our study exhibited resistance to multiple other clinically relevant antimicrobials, consistent with many reports documenting extensive multidrug resistance in CRKP worldwide [30,31]. Moreover, the literature indicates that colistin MICs tend to be higher in isolates harboring chromosomal mutations affecting regulatory genes compared to isolates with plasmid-mediated resistance mechanisms (*mcr* genes) [35], highlighting distinct resistance dynamics between genomic and plasmid sources.

All the colistin-resistant CRKP isolates belonged to diverse sequence types (ST11, ST101, ST147, ST2502, and ST4870), reflecting substantial genomic plasticity. All carried the *bla*_OXA-48_ carbapenemase gene, while most also harbored ESBL genes such as *bla*_CTX-M-15_; additionally, two strains carried *bla*_NDM-1_. This resistance gene profile aligns with patterns observed in other studies, where high-risk clones like ST11 and ST147 are key vectors of *bla*_OXA-48_ and *bla*_NDM-1_, and the regional distribution of these enzymes can vary significantly [36,37,38]. Co-occurrence of OXA-48 with ESBLs, particularly *bla*_CTX-M-15_, is also commonly reported [36]. Moreover, the detection of plasmid-mediated quinolone resistance genes (*qnrS1*, *qnrB1*, and *aac(6′)-Ib-cr*) is consistent with previous studies showing their frequent association with ESBL- and carbapenemase-producing *K. pneumoniae*, especially in NDM- and KPC-positive strains [39]. Importantly, none of our isolates carried plasmid-mediated *mcr* genes, in line with surveillance data indicating that *mcr*-driven colistin resistance remains relatively uncommon in *K. pneumoniae* [30,40].

ERIC-PCR grouped the five colistin-resistant CRKP isolates into four pulsotypes and MLST identified five sequence types (ST11, ST101, ST147, ST2502 and ST4870), including internationally disseminated high-risk lineages (ST11, ST101 and ST147). These successful clones are frequently associated with carbapenemase acquisition and nosocomial spread, and ST101 has been increasingly recognized as an emerging high-risk CRKP lineage in multiple settings. The polyclonal structure observed here, together with recovery from different wards and across multiple years, is more consistent with multiple independent acquisition/selection events occurring within an endemic CRKP background than with a single clonal outbreak. Nevertheless, because several isolates were recovered from critical-care units over a short period, unrecognized within-ward transmission cannot be excluded. Higher-resolution genomic comparisons coupled with patient-movement data would be required to distinguish repeated introductions from silent transmission and to better inform targeted infection-control interventions.

To provide local genomic context for the five colistin-resistant isolates, we expanded the phylogenetic reconstruction by including a contemporaneous comparator panel of 19 colistin-susceptible carbapenem-resistant *K. pneumoniae* isolates (CS-CRKP01 to CS-CRKP19). In this broader dataset, the colistin-resistant isolates did not form a single monophyletic cluster but were distributed across distinct sequence types and interspersed with susceptible isolates (including within the predominant ST147 background), supporting multiple emergence events rather than dissemination of one colistin-resistant clone. The accompanying heatmap further shows that key resistance determinants—carbapenemases (*bla*_OXA-48_ and/or *bla*_NDM-1_), ESBL *bla*_CTX-M-15_ and additional β-lactamases (*bla*_TEM-1_, *bla*_SHV-11_, *bla*_OXA-1_, *bla*_DHA-1_)—and PMQR markers (*qnrB1*, *qnrS1*, *aac*(6′)-Ib-cr) were largely shared between colistin-resistant and colistin-susceptible CRKP. Together, these findings indicate that colistin resistance in our setting emerged on established multidrug-resistant backgrounds rather than being associated with a distinctive β-lactamase/PMQR profile; virulence-associated genes were also broadly conserved, with *rmp* and *kfu* restricted to a single isolate (CRKP3).

Although porin loss primarily contributes to reduced susceptibility to β-lactams rather than to polymyxins, we included OmpK35/OmpK36 characterization to provide a more complete picture of the multidrug-resistant background in which chromosomal colistin resistance arises. We did not investigate other potential contributors to resistance such as efflux pumps or penicillin-binding proteins, which represent an additional limitation of our study.

This qualitative pattern was supported by densitometric analysis normalized to OmpA, confirming reduced/undetectable OmpK35 in CRKP1, CRKP2, and CRKP3 and reduced/undetectable OmpK36 in CRKP4 and CRKP5. While SDS-PAGE densitometry remains a relative measure, normalization to OmpA provides an objective comparison across lanes and supports the observed pattern of selective porin reduction.

Carbapenem resistance in *K. pneumoniae* is frequently associated with loss or functional alteration of the major porins OmpK35 and OmpK36, often caused by insertion sequences or mutations leading to truncated, non-functional proteins, as demonstrated in this study. These findings are consistent with Tunisian reports that highlight porin loss, in combination with carbapenemase production, as a key mechanism of resistance [14,26]. These data underscore the multifactorial nature of CRKP resistance, where both enzymatic degradation and impaired antibiotic influx through porin disruption synergistically reduce treatment efficacy. In this context, we further explored a plausible regulatory mechanism for porin loss in CRKP1. In silico promoter analysis suggested a transcriptional impact of the upstream ISKpn14 insertion, BPROM predicted a markedly weaker σ70-like promoter signal in CRKP1 compared with the reference upstream region, consistent with disruption of the native promoter architecture upstream of *ompK35* and reduced OmpK35 production. While such computational predictions do not replace functional validation, they provide supportive evidence linking the IS insertion to the observed loss of *OmpK35* in the outer membrane protein profile.

The virulence gene profile, dominated by *entB*, *ybtS*, and *mrkD*, with *rmpA* and *kfu* detected only in one isolate, suggests that colistin-resistant CRKP in our cohort generally retain baseline virulence and fitness traits but only occasionally carry markers more typically associated with hypervirulence. Beyond pathogenicity per se, these loci may also contribute to persistence and environmental adaptation in the hospital setting: *mrkD* (type 3 fimbrial adhesin) is linked to adherence and biofilm formation on abiotic surfaces, whereas siderophore-related genes such as *entB* (enterobactin) and *ybtS* (yersiniabactin) support growth under iron limitation and may enhance survival under host- and stress-associated conditions. The coexistence of these traits with a multidrug-resistant background is consistent with strains that are well adapted to the nosocomial environment, potentially facilitating long-term maintenance and silent dissemination of high-risk CRKP lineages, while acknowledging that gene presence alone does not prove increased virulence.

The absence of plasmid-mediated *mcr* genes in our isolates is consistent with prior studies suggesting that chromosomal mutations are the dominant mechanism of colistin resistance in clinical *K. pneumoniae* strains [40]. All isolates harbored disruptive mutations in the *mgrB*-*phoPQ* regulatory system, including insertions, deletions, or substitutions causing premature stop codons or frameshifts. One isolate (CRKP2) lacked *mgrB* alterations but presented inactivating mutations in both *phoP* and *phoQ*, suggesting that colistin resistance can emerge independently of *mgrB* disruption. Importantly, although our study did not detect insertion sequences in *mgrB*, previous studies have reported that *IS* elements are the most frequent mechanism of *mgrB* inactivation globally [41,42]. These sequences, such as ISKpn14 or IS5-like elements, often insert into the coding region or promoter of *mgrB*, thereby silencing its expression and conferring resistance [42]. Our findings support previous reports indicating that while *mgrB* inactivation is a predominant mechanism, mutations in other components of the PhoPQ system can also confer resistance [35,43].

Importantly, these alterations were not observed in a contemporaneous set of colistin-susceptible CRKP isolates, supporting that they are associated with colistin resistance rather than representing lineage-specific polymorphisms.

Across our isolates, the mutations detected in the PhoPQ pathway can be grouped into two broad categories. (i) Disruptive variants (frameshifts and/or premature stop codons), which are strongly compatible with a functional impact on the pathway, and (ii) non-truncating, non-synonymous substitutions, for which the functional effect on signaling and downstream lipid A remodeling cannot be inferred from sequence data alone. In our dataset, disruptive events included frameshift/stop mutations in *mgrB* (CRKP1, CRKP3, CRKP4 and CRKP5) and truncating alterations in PhoP/PhoQ (e.g., PhoP stop at aa7 and PhoQ stop at aa4 in CRKP4; PhoQ stops at aa175, aa41 and aa207 in CRKP1, CRKP2 and CRKP5, respectively). Conversely, several isolates carried multiple amino-acid substitutions in PhoP (and/or PhoQ) without protein truncation, whose contribution to colistin resistance cannot be determined without functional testing. Accordingly, although the overall mutational pattern is consistent with functional impairment of the PhoPQ regulatory cascade, classically linked to *arn*/*pmr* operon activation and L-Ara4N/PEtN lipid A remodeling that reduces colistin binding, we were not able to perform functional studies (complementation assays, lipid A profiling, or gene-expression analysis); therefore, we cannot definitively distinguish neutral allelic variation from causal substitutions among the non-truncating changes.

Our data support that chromosomal mechanisms represent the predominant determinants associated with colistin resistance in this Tunisian CRKP collection and that these occur in diverse clonal lineages, both globally disseminated and locally emerging. These observations underline the importance of genomic surveillance to monitor the evolution and spread of resistance in critical-care settings.

We next evaluated whether the colistin-resistant phenotype was maintained in the absence of antibiotic pressure. After seven serial passages without colistin, MICs remained unchanged, supporting short-term stability of the resistance phenotype in our isolates. This observation is consistent with prior work showing that clinical colistin-resistant *K. pneumoniae* can persist during serial passaging in colistin-free media, including KPC-producing lineages where resistant isolates remained stable over repeated daily transfers without colistin [34]. In vivo models have also supported stability of *mgrB*-associated colistin resistance following passage through an infection setting, suggesting that resistance can be maintained beyond purely in vitro conditions [44].

However, stability is not universal across Gram-negative species or resistance mechanisms. In Pseudomonas aeruginosa, colistin-resistant phenotypes have been reported to revert to susceptibility after serial passaging in drug-free medium (over ~20–28 passages), consistent with genetic reversion and fitness restoration when selective pressure is removed [45]. Likewise, colistin-susceptible revertants have been described in *Acinetobacter baumannii* following passage in the absence of colistin [46]. In addition, when colistin resistance is plasmid-mediated, repeated passaging without colistin may facilitate plasmid elimination and loss of resistance determinants, resulting in decreased colistin MICs [47]. Collectively, these data suggest that the persistence of colistin resistance depends on the underlying genetic basis and associated fitness costs; in our CRKP isolates, the lack of MIC change over 7 days supports a stable phenotype over this timeframe. Moreover, targeted resequencing of *mgrB* after day 7 revealed no sequence changes relative to baseline, suggesting that *mgrB*-mediated resistance determinants were genetically stable over this timeframe.

### Limitations

This study has several limitations. First, only five colistin-resistant CRKP isolates were investigated, all recovered from a single tertiary-care hospital, which limits the generalizability of our findings to other Tunisian or regional healthcare settings. Second, the isolates were collected between 2010 and 2015; they therefore reflect an earlier therapeutic context in which colistin was more frequently used against CRKP and may not fully capture the current epidemiology in the era of newer agents such as β-lactam/β-lactamase inhibitor combinations and cefiderocol. Third, our molecular analysis focused on a limited set of chromosomal loci (*mgrB*, *phoP*, *phoQ*), and we did not explore other pathways that may contribute to colistin or carbapenem resistance, such as PmrAB/CrrAB two-component systems, efflux pumps or penicillin-binding proteins. Fourth, we did not have access to whole-genome sequencing for these isolates, which would have provided a more comprehensive view of resistance determinants and mobile genetic elements. Finally, we did not perform functional assays to validate the impact of the identified mutations on lipid A modification or colistin susceptibility; as a result, the causal role of individual amino acid changes in PhoP/PhoQ remains inferred rather than experimentally demonstrated. Although Table 5 provides a structured genotype–phenotype synthesis, it does not substitute for functional validation such as lipid A profiling (e.g., MALDI-TOF) or gene-expression analyses of PhoPQ-regulated targets.

We did not investigate the *crrA/crrB* two-component system; thus, we cannot exclude its potential contribution to colistin resistance in some isolates. Future work should include targeted PCR/sequencing of the crrA/crrB two-component system to clarify its potential contribution to colistin resistance in these isolates.

Because sequencing of *ompK35/ompK36* was not performed in colistin-susceptible internal controls, porin findings are presented as contextual features of the MDR background.

## 5. Conclusions

Our findings show that colistin resistance in CRKP isolates from a Tunisian hospital was consistently associated with inactivating mutations in the MgrB–PhoPQ pathway in the absence of *mcr* genes. These alterations were observed in both globally disseminated and locally emerging sequence types, underscoring the diversity and adaptability of CRKP in clinical environments. Although newer therapeutic options for CRKP have become available in recent years, colistin remains an important component of treatment in many settings, particularly where access to these agents is limited. Continuous surveillance and molecular characterization are therefore essential to detect the emergence of resistance and to inform infection control and treatment strategies.

## Figures and Tables

**Table 1:** Clinical characteristics, treatment, and outcomes of patients infected with colistin-resistant CRKP.

Patient	Strain ID	Ward	Specimen Type	Isolation Date	Age (Years)/Gender	Underlying Disease	Antibiotic Treatment	Outcome
1	CRKP1	ICU ^1^	Pulmonary	2013	27/M ^2^	Respiratory distress	Tigecycline, Gentamicin, Ceftazidime	Improved
2	CRKP2	Orthopedics	Catheter	2015	84/F ^3^	Polytraumatism	Amoxicillin + clavulanate	Improved
3	CRKP3	ICU ^1^	Catheter	2015	75/M ^2^	Respiratory distress	Vancomycin, Imipenem, Rifampin, Amikacin, Colistin, Fosfomycin	Died
4	CRKP4	ICU ^1^	Pulmonary	2015	32/M ^2^	Polytraumatism	Gentamicin, Imipenem, Colistin, Vancomycin, Ciprofloxacin	Improved
5	CRKP5	ICU	Wound	2015	24/M ^2^	Polytraumatism	Amoxicillin + clavulanate, Gentamicin, Imipenem, Fosfomycin, Colistin, Ciprofloxacin	Improved

^1^ ICU, Intensive Care Unit; ^2^ M, Male; ^3^ F, Female.

**Table 2:** Antibiotic resistance profiles of colistin-CRKP isolates.

Strain	Resistance Patterns	Phenotypic Assays				MICs *																
		MHT ^1^	CIM ^2^	EDTA ^3^	PBA ^4^	E-Test Strips			VITEK													
						ETP ^5^	IMP ^6^	MEM ^7^	AMP ^8^	AMC ^9^	TZP ^10^	CXM ^11^	FOX ^12^	CTX ^13^	CAZ ^14^	FEP ^15^	AMK ^16^	GM ^17^	NAL ^18^	CIP ^19^	TGC ^20^	SXT ^21^
CRKP1	AMP ^8^, AMC ^9^, TZP ^10^, CXM ^11^, FOX ^12^, CTX ^13^, CAZ ^14^, FEP ^15^, AMK ^16^, NAL ^18^, CIP ^19^, SXT ^21^, CRO ^22^	+	+	-	-	>32	24	24	≥32	≥32	≥128	≥64	≥64	≥64	≥64	≤1	16	≤1	≥32	≥4	1	≥320
CRKP2	AMP ^8^, AMC ^9^, TZP ^10^, CXM ^11^, FOX ^12^, CTX ^13^, CAZ ^14^, FEP ^15^, AMK ^16^, GM ^17^, NAL ^18^, CIP ^19^, SXT ^21^, CRO ^22^	+	+	-	-	>32	4	12	≥32	≥32	≥128	≥64	≥64	≥64	≥64	≥64	16	≥16	≥32	≥4	2	40
CRKP3	AMP ^8^, AMC ^9^, TZP ^10^, CXM ^11^, FOX ^12^, CTX ^13^, CAZ ^14^, FEP ^15^, AMK ^16^, GM ^17^, NAL ^18^, CIP ^19^, TGC ^20^, SXT ^21^, CRO ^22^	+	+	-	-	>32	4	6	≥32	≥32	≥128	≥64	≥64	≥64	16	≥64	≥64	≥16	≥32	≥4	2	≥320
CRKP4	AMP ^8^, AMC ^9^, TZP ^10^, CXM ^11^, FOX ^12^, CTX ^13^, CAZ ^14^, FEP ^15^, GM ^17^, NAL ^18^, CIP ^19^, TGC ^20^, SXT ^21^, CRO ^22^	+	+	+	-	32	12	16	≥32	≥32	≥128	≥64	≥64	≥64	≥64	16	8	≥16	≥32	≥4	4	≥320
CRKP5	AMP ^8^, AMC ^9^, TZP ^10^, CXM ^11^, FOX ^12^, CTX ^13^, CAZ ^14^, FEP ^15^, GM ^17^, NAL ^18^, CIP ^19^, TGC ^20^, SXT ^21^, CRO ^22^	+	+	+	-	2	2	1	≥32	≥32	≥128	≥64	≥64	≥64	≥64	≥64	8	≥16	≥32	≥4	4	≥320

^1^ MHT, Modified Hodge Test; ^2^ CIM, Carbapenem Inactivation Method; ^3^ EDTA, Ethylene Diamine Tetraacetic Acid; ^4^ PBA, Phenyl Boronic Acid; ^5^ ETP, Ertapenem; ^6^ IMP, Imipenem; ^7^ MEM, Meropenem; ^8^ AMP, Ampicillin; ^9^ AMC, Amoxicillin-clavulanic acid; ^10^ TZP, piperacillin–tazobactam; ^11^ CXM, cefuroxime; ^12^ FOX, cefoxitin; ^13^ CTX, cefotaxime; ^14^ CAZ, ceftazidime; ^15^ FEP, cefepime; ^16^ AMK, Amikacin; ^17^ GM, Gentamicin; ^18^ NAL, nalidixic acid; ^19^ CIP, ciprofloxacin; ^20^ TGC, tigecycline; ^21^ SXT, sulfamethoxazole/trimethoprim; ^22^ CRO, ceftriaxone; * MICs were determined according to EUCAST guidelines (mg/L).

**Table 3:** Porin alterations, nucleotide and protein mutations in *ompK35* and *ompK36* among colistin-resistant CRKP.

Isolate	SDS-PAGE (OmpK35)	SDS-PAGE (OmpK36)	Genetic Alteration Linked to the Missing Porin *	Predicted Consequence (for the Missing Porin)	Interpretation (Consistent with Missing Porin on SDS-PAGE)
CRKP1	Not detected	Detected	IS1 insertion at −852	Promoter/5′ region disruption	Likely transcriptional downregulation → OmpK35 not detected
CRKP2	Not detected	Detected	Δ6 nt (CACCAA) at nt 137; +G at nt 166	Frameshift → premature stop; truncated OmpK35 (314 aa)	Loss-of-function → OmpK35 not detected
CRKP3	Not detected	Detected	C156T; ΔG at nt 184	Frameshift → premature stop; truncated OmpK35 (62 aa)	Loss-of-function → OmpK35 not detected
CRKP4	Detected	Not detected	Multiple nonsynonymous substitutions; in-frame insertion of 9 nt (CTGTCTCCT) at nt 550	Altered OmpK36 sequence	Likely structural alteration and/or reduced detection → OmpK36 not detected
CRKP5	Detected	Not detected	+G at nt 65; +T at nt 129	Frameshift → premature stop; truncated OmpK36 (22 aa)	Loss-of-function → OmpK36 not detected

* Positions are numbered relative to the start codon (ATG = +1); negative values indicate upstream positions. IS, insertion sequence; nt, nucleotide(s); aa, amino acid(s); Δ, deletion; +, insertion; →, leads to.

**Table 4:** Summary of *mgrB*, *phoP* and *phoQ* mutations and predicted effects in colistin-resistant CRKP isolates.

Strain	*mgrB* Mutations	Predicted Effect	*phoP* Mutations	Predicted Effect	*phoQ* Mutations	Predicted Effect
CRKP1	ΔT132	Frameshift →Altered protein sequence	C29T; C31A; C363T	AA substitutions	C219T; InsA486; InsGC, 498-499	Premature stop codon at AA 175
CRKP2	None	Intact MgrB	G57C; T457G; G554C	AA substitutions	T32G; C35G; A36C; T37A; T38G; ΔC48; InsA58; InsAA65-66; ΔC91	Premature stop codon at AA 41
CRKP3	ΔT132	Frameshift → altered protein sequence	A471T; C510A; C537T	AA substitutions	ΔA5; G29C; T130A; ΔC611	Frameshift → altered protein sequence
CRKP4	Multiple point mutations; ΔA42, ΔCC48-49	Premature stop at AA19	InsA7; C32T; C47A; C112A; InsA143	Premature stop codon at AA7	C10T; T11G; G12A; T14C; G15T; G17T; C19T; A20T; ΔT34; ΔGCC105-106-107	Premature stop codon at AA 4
CRKP5	ΔT132	Frameshift → altered protein sequence	T17A; C31G	AA substitutions	C219T; C512A; C517A; ΔG527; InsT538	Premature stop codon at AA 207

Δ, nucleotide deletion; Ins, nucleotide insertion; AA, amino acid.

**Table 5:** Genotype–phenotype summary: colistin MICs and predicted-impact mutations in the MgrB–PhoPQ pathway.

Isolate	Colistin MIC (BMD; mg/L)	*mgrB* Alteration (Type; Key Position)	*phoP* Alteration (Type)	*phoQ* Alteration (Type; Key Position)	Predicted Pathway-Level Impact (Descriptive)	Number of Disrupted Components (0–3) *
CRKP1	32	ΔT132; frameshift (disruptive)	Missense substitutions (non-disruptive)	Premature stop at aa175 (truncation; disruptive)	Disruptive alterations affecting *mgrB* and *phoQ*; consistent with functional impairment of the PhoPQ pathway	2
CRKP2	16	Wild-type	Missense substitutions (non-disruptive)	Premature stop at aa41 (early truncation; disruptive)	Disruptive alteration in *phoQ* with intact *mgrB*; functional impairment of the PhoPQ pathway	1
CRKP3	8	ΔT132; frameshift (disruptive)	Missense substitutions (non-disruptive)	Frameshift (disruptive)	Disruptive alterations affecting *mgrB* and *phoQ*; functional impairment of the PhoPQ pathway	2
CRKP4	8	Premature stop at aa19 (early truncation; disruptive)	Premature stop at aa7 (early truncation; disruptive)	Premature stop at aa4 (very early truncation; disruptive)	Multiple very early truncations in *mgrB/phoP/phoQ*; highly disruptive profile within the PhoPQ system	3
CRKP5	8	ΔT132; frameshift (disruptive)	Missense substitutions (non-disruptive)	Premature stop at aa207 (truncation; disruptive)	Disruptive alterations affecting *mgrB* and phoQ; functional impairment of the PhoPQ pathway	2

* Counted across *mgrB*, *phoP* and *phoQ* (0–3). Disruption was defined as frameshift or premature stop codon (and IS interruption when present). Full nucleotide-level details are provided in Table 4; BMD, Broth Microdilution; MIC, Minimum Inhibitory Concentration; Δ, nucleotide deletion; aa: amino acid.

## Data Availability

The data that support the findings of this study are available from the corresponding author upon reasonable request.

## Supplementary Materials

The following supporting information can be downloaded at: https://www.mdpi.com/article/10.3390/idr18030042/s1, Figure S1. Sodium dodecyl sulfate-polyacrylamide gel electrophoresis analysis of OMPs from the five colistin-resistant CRKP isolates. Figure S2. Annotated schematic of the upstream region of ompK35 in the reference sequence (Wild Type WT, K. pneumoniae NTUH-K2044) and in CRKP1. Table S1: Densitometric quantification of outer membrane porins.

## Abbreviations

The following abbreviations are used in this manuscript:

AMR	Antimicrobial resistance
CIM	Carbapenem Inactivation Method
CLSI	Clinical and Laboratory Standards Institute
CRKP	Carbapenem-resistant *Klebsiella pneumoniae*
DDST	Double Disk Synergy Test
EDTA	Ethylenediaminetetraacetic acid
ERIC-PCR	Enterobacterial Repetitive Intergenic Consensus Polymerase Chain Reaction
ESBL	Extended-spectrum β-lactamase
EUCAST	European Committee on Antimicrobial Susceptibility Testing
ICU	Intensive Care Unit
LPS	Lipopolysaccharide
MALDI-TOF	Matrix-Assisted Laser Desorption/Ionization–Time of Flight
MDR	Multidrug-resistant
MEGA	Molecular Evolutionary Genetics Analysis
MIC	Minimum inhibitory concentration
MLST	Multilocus sequence typing
NDM	New Delhi Metallo-β-lactamase
OMP	Outer membrane protein
PBA	Phenylboronic acid
PMQR	Plasmid-mediated quinolone resistance
SDS-PAGE	Sodium dodecyl sulfate–polyacrylamide gel electrophoresis
ST	Sequence type
TCS	Two-component system
