# Supplementary material for: Chromosomal Mechanisms of Colistin Resistance in Clinical Isolates of Carbapenem-Resistant Klebsiella pneumoniae from a Tunisian Tertiary-Care Hospital
Source: Infect Dis Rep. 2026 May 1;18(3):42. doi: 10.3390/idr18030042 (PMC13214794; doi:10.3390/idr18030042)
Supplement: Supplementary file 1 [file idr-18-00042-s001.zip › idr-4026327-supplementary.pdf]

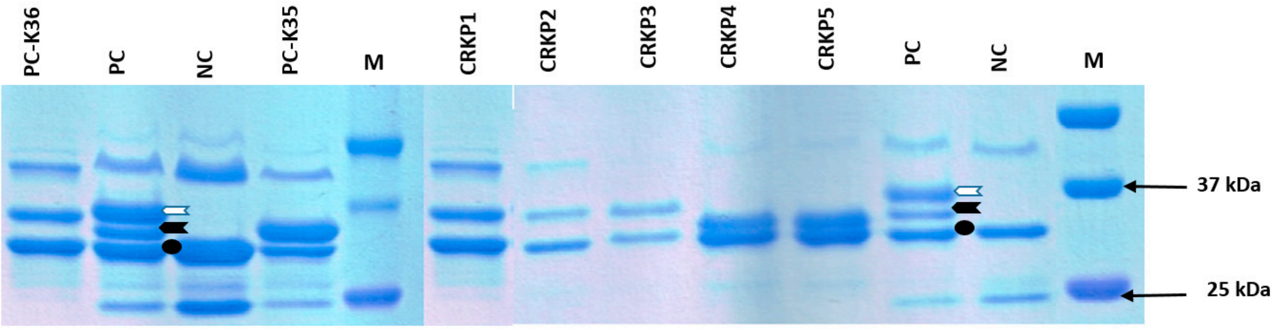

Figure S1. Sodium dodecyl sulfate-polyacrylamide gel electrophoresis analysis of OMPs from the five colistin-resistant CRKP isolates. Lanes (left to right): PC-K36, PC, NC, PC-K35, M, CRKP1–CRKP5, PC, NC, M. M, Molecular size markers (in Kilodaltons); PC, Positive Control producing both OmpK35 and OmpK36 (K. pneumoniae SD8); NC, Negative Control producing neither OmpK35 nor OmpK36 (*K. pneumoniae* CSUB10R); PC-K36, Positive Control producing only OmpK36 (reference strain *K. pneumoniae* NTUH-K2044); PC-K35, Positive Control producing only OmpK35 (reference strain *K. pneumoniae KCTC2242*). Symbols: White arrow, OmpK36; black arrow, OmpK35; solid circle, OmpA.

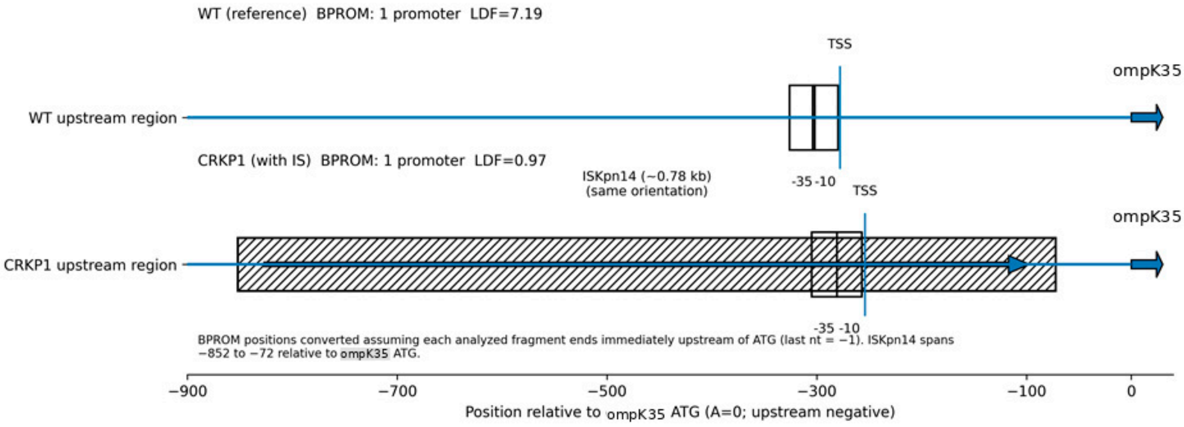

Figure S2. Annotated schematic of the upstream region of *ompK35* in the reference sequence (Wild Type WT, *K. pneumoniae* NTUH-K2044) and in CRKP1. In CRKP1, ISKpn14 (~0.78 kb; IS1-family) spans −852 to −72 relative to the A of the ompK35 ATG and is inserted in the same orientation as *ompK35*. Putative housekeeping (σ70/RpoD) promoter elements were predicted using BPROM (SoftBerry). For the WT upstream fragment (length 460 bp), BPROM predicted one promoter (LDF 7.19) with −35 and −10 elements and a predicted transcription start site (TSS). For the corresponding CRKP1 upstream fragment (length 460 bp), BPROM predicted a much weaker promoter signal (LDF 0.97), including a very low −35 score. Positions of −35/−10/TSS are shown relative to ATG for visualization by converting BPROM coordinates under the assumption that each analyzed fragment ends immediately upstream of ATG (−1).

Table S1. Densitometric quantification of outer membrane porins.

Sample	Phenotype	OmpA area (AU)	OmpK35 area (AU)	OmpK36 area (AU)	OmpK35/OmpA	OmpK36/OmpA	OmpK35 (% of PC-K35)	OmpK36 (% of PC-K36)
PC-K36	OmpK36+ OmpK35−	6335.255	ND	3543.355	ND	0.559	ND	100.0
PC	OmpK36+ OmpK35+	2448.698	781.213	2051.820	0.319	0.838	14.5	149.8
NC	OmpK36− OmpK35−	9603.104	ND	ND	ND	ND	ND	ND
PC-K35	OmpK36− OmpK35+	1413.092	3104.234	ND	2.197	ND	100.0	ND
CRKP1	OmpK36+ OmpK35−	5492.012	ND	2903.527	ND	0.529	ND	94.5
CRKP2	OmpK36+ OmpK35−	2287.698	ND	896.163	ND	0.392	ND	70.0
CRKP3	OmpK36+ OmpK35−	1182.335	ND	1235.577	ND	1.045	ND	186.8
CRKP4	OmpK36− OmpK35+	657.577	148.556	ND	0.226	ND	10.3	ND
CRKP5	OmpK36− OmpK35+	601.456	174.385	ND	0.290	ND	13.2	ND

PC, positive control expressing both OmpK35 and OmpK36 (K. pneumoniae SD8); NC, negative control lacking detectable OmpK35 and OmpK36 (OmpA present; K. pneumoniae CSUB10R); PC-K36, control expressing OmpK36 only (OmpA present; K. pneumoniae NTUH-K2044; accession AP006725); PC-K35, control expressing OmpK35 only (OmpA present; K. pneumoniae KCTC2242; accession CP002910). ND, not detected. AU, arbitrary units (integrated density; area under the densitometry peak measured in Fiji/ImageJ after background subtraction). Marker lanes were excluded. OmpA served as a loading control for normalization. Percent expression was calculated relative to porin-specific controls (PC-K35 for OmpK35; PC-K36 for OmpK36) set to 100% (values may exceed 100%).
